# A comparison of thyroidal protection by iodine and perchlorate against radioiodine exposure in Caucasians and Japanese

**DOI:** 10.1007/s00204-021-03065-5

**Published:** 2021-05-18

**Authors:** A. Rump, S. Eder, C. Hermann, A. Lamkowski, M. Kinoshita, T. Yamamoto, M. Abend, N. Shinomiya, M. Port

**Affiliations:** 1 Bundeswehr Institute of Radiobiology, Neuherberg Str. 11, 80937 Munich, Germany; 2 Japan Self Defense Forces National Defense Medical College Research Institute, Tokorozawa, Japan; 3 Japan Ground Self Defense Force NBC Countermeasure Medical Unit, Tokyo, Japan

**Keywords:** Medical NRBC protection, Nuclear and radiological emergency, Radioiodine, Iodine blockade, Perchlorate, Nutrition, Ethnopharmacology

## Abstract

Radioactive iodine released in nuclear accidents may accumulate in the thyroid and by irradiation enhances the risk of cancer. Radioiodine uptake into the gland can be inhibited by large doses of stable iodine or perchlorate. Nutritional iodine daily intake may impact thyroid physiology, so that radiological doses absorbed by the thyroid as well as thyroid blocking efficacy may differ in Japanese with a very rich iodine diet compared to Caucasians. Based on established biokinetic–dosimetric models for the thyroid, we derived the parameters for Caucasians and Japanese to quantitatively compare the effects of radioiodine exposure and the protective efficacy of thyroid blocking by stable iodine at the officially recommended dosages (100 mg in Germany, 76 mg in Japan) or perchlorate. The maximum transport capacity for iodine uptake into the thyroid is lower in Japanese compared to Caucasians. For the same radioiodine exposure pattern, the radiological equivalent thyroid dose is substantially lower in Japanese in the absence of thyroid blocking treatments. In the case of acute radioiodine exposure, stable iodine is less potent in Japanese (ED_50_ = 41.6 mg) than in Caucasians (ED_50_ = 2.7 mg) and confers less thyroid protection at the recommended dosages because of a delayed responsiveness to iodine saturation of the gland (Wolff–Chaikoff effect). Perchlorate (ED_50_ = 10 mg in Caucasians) at a dose of 1000 mg has roughly the same thyroid blocking effect as 100 mg iodine in Caucasians, whereas it confers a much better protection than 76 mg iodine in Japanese. For prolonged exposures, a single dose of iodine offer substantially lower protection than after acute radioiodine exposure in both groups. Repetitive daily iodine administrations improve efficacy without reaching levels after acute radioiodine exposure and achieve only slightly better protection in Japanese than in Caucasians. However, in the case of continuous radioiodine exposure, daily doses of 1000 mg perchlorate achieve a high protective efficacy in Caucasians as well as Japanese (> 0.98). In Caucasians, iodine (100 mg) and perchlorate (1000 mg) at the recommended dosages seem alternatives in case of acute radioiodine exposure, whereas perchlorate has a higher protective efficacy in the case of longer lasting radioiodine exposures. In Japanese, considering protective efficacy, preference should be given to perchlorate in acute as well as prolonged radioiodine exposure scenarios.

## Introduction

Nuclear fission reactions generate several hundred radionuclides, most of them with very short decay half-lives (Buddemeier 2018). These are responsible for the extremely high activities in the mushroom clouds after the detonation of nuclear weapons and the very high dose rates emanating from early fallout. External irradiation is at first the key issue. The quantitative predominance of these very short-lived nuclides also explains the rapid decay of activity (reduction to 10% of the initial activity after 7 h as a rule of thumb). In the further course, radionuclides having medium or longer decay half-lifes remain and will be spread in the atmosphere depending on meteorological conditions, causing regional and/or global fallout. With increasing distance from the detonation point and dilution of the activity, the impact of external irradiation decreases and the risks emanating from the incorporation of radionuclides and internal contamination relatively increases (Simon et al. 2006). In a nuclear reactor, the fission reaction is basically the same, but processes are more protracted in time, and the mechanisms involved in a power plant accident may vary (e.g., power surge accident in Chernobyl, coolant deficiency accident in Fukushima) (Imanaka et al. 2015). Therefore, the radioactive mixtures released may quantitatively differ (Wohni 1995; Imanaka et al. 2015).

Radioactive cesium, strontium and iodine are among the nuclides generated with a higher yield and associated with particular health hazards (Chabot 2016; Buddemeier 2018). Radioactive cesium is relatively volatile and has a long decay half-life (for cesium-137, physical T_1/2_: 30 years; biological half-life: 70–130 days). In the body, it behaves similarly to potassium. Although it is possible to speed up cesium elimination out of the body by Prussian Blue (Rump et al. 2016), in the case of environmental contamination on a large scale, the only practical and meaningful strategy will rely on limiting public exposure and controlling the radioactivity content of the food produced in the affected area. Strontium and in particular strontium-90 with a long physical decay half-life (28 years) is particularly critical as similar to calcium it accumulates in bones, where it remains for an extended period of time (biological half-life about 5.5 years). Fortunately, it is much less volatile than cesium, so that after a nuclear incident the contaminated area remains smaller than for cesium, as observed after the power plant accident in Chernobyl. Radioiodine, in particular iodine-131 as the most important nuclide, differ in so far from cesium and strontium as it shows a very high volatility and thus spreads easily over larger areas, but it has a relatively short physical decay half-life of 8 days (Geoffroy et al. 2000; Chabot 2016; Rump et al. 2019). Therefore, it is not a radionuclide causing long term environmental contamination issues. Nevertheless, it poses a relevant health hazard after nuclear incidents as it is easily absorbed in the body by inhalation or ingestion and rapidly concentrates in the thyroid inducing an irradiation of the gland that may destroy the tissue leading to hypothyroidism and through stochastic radiation damages enhances the risk of thyroid cancer occurrence (Geoffroy et al. 2000; Rump et al. 2019). An increase of thyroid cancers has been observed in Ukraine, Belorussia and Russia following the Chernobyl accident in the population having been exposed to radioiodine in childhood (Lomat 1997; Henriksen 2014). Even in the case of early fallout exposure, at the difference of other tissues, most of the radiological dose absorbed by the thyroid seems to be due to internal contamination of the gland by incorporated radioiodine, and not by external irradiation, as could be shown by examinations of the affected inhabitants following the Castle Bravo thermonuclear test accident on the Marshall Islands in 1954 (Simon et al. 2010).

As for the time being there is no treatment to effectively speed up the secretion of radioiodine integrated in thyroidal hormones from the gland, the only effective way to achieve protection is by blocking the net uptake of radioiodine from serum and its accumulation in the thyrocytes. This can be achieved by the intake of a large, but still not toxic amount of stable iodine (100 mg) and this is the procedure recommended by the WHO (2017) and most national nuclear safety regulation authorities (ASN 2008; SSK 2018). Two protective mechanisms are involved (Geoffroy et al. 2000; Rump et al. 2019; Eder et al. 2020): as iodide is transported through the basolateral membrane of the thyrocytes into the cells by a saturable active carrier (sodium–iodide-symporter, NI-symporter) (Darrouzet et al. 2014), stable iodine will compete with radioiodine and because it is in a large excess amount markedly reduce radioiodine uptake that at the same time is subject to renal elimination. A second less elucidated mechanism sometimes described as “saturation” (Wolff–Chaikoff effect) leads to a complete block of net iodine uptake (Wolff and Chaikoff 1948). This effect, however, is only transient (24–48 h) (Geoffroy et al. 2000). Although the administration of stable iodine is for the time being the standard to achieve “iodine blockade” in the case of radioiodine exposure (WHO 2017), it seems also possible to use other antithyroidal agents like perchlorate, provided it is administered at equieffective dosages (Harris et al. 2009; Hänscheid et al. 2011; Eder et al. 2020). Considering the adverse effects, it does not seem that the preference given to stable iodine is so obvious as sometimes stated, in particular in certain vulnerable populations for whom a particular sensitivity to the Wolff–Chaikoff effect has been reported (e.g., the fetus).

A particular issue relates to the repetitive administrations of protective agents. In most recommendations, a single dose of stable iodine is advised, although it is acknowledged that in the case of prolonged radioiodine exposure that cannot be avoided, repetitive dosages may become necessary. Simulations show that a single stable iodine dose in the case of a continuous radioiodine exposure is associated with a much lower efficacy than in the case of an acute exposure (Rump et al. 2019; Eder et al. 2020). There is, however, a lack of concrete recommendations in official documents on this point. Considering the nuclear plant accidents in Chernobyl and Fukushima, in both cases radioactivity releases occurred over roughly 10 days, although amounts largely differed as well as the pattern of the time course (Imanaka et al. 2015). So, past experiences indicate that a longer lasting activity release must be expected, and therefore, therapeutic principles should be established to help physicians and authorities how to behave in such rare emergencies.

Considering the mechanism of action, it is obvious that stable iodine must be in excess to radioiodine to achieve thyroidal protection. However, the precise dosage may be a subject of discussion. In most countries, the official recommendations for adults amount to 76 mg iodine (100 mg potassium iodide) (e.g., in Japan) (Yoshida et al. 2014) or 100 mg iodine (130 mg potassium iodide) (e.g., in Germany) (SSK 2018; European Commission 2010). In the literature, it is sometimes hypothesized that lower dosages might be sufficient (Kunii et al. 2016). It is also acknowledged that the sensitivity of the thyroid towards radioiodine as well as the efficacy of a particular stable iodine dosage for protection may depend on the function of the gland (euthyroid, hypothyroid or hyperthyroid state). This is reflected by the huge variability of the thyroidal clearance for iodide and the extraction rate out of the plasma ranging from 10 to 40% in euthyroid persons (Kovari et al. 1994; Geoffroy et al. 2000; Verger et al. 2001) up to 80% in patients with Grave’s hyperthyroidism. Another factor that may affect the sensitivity to radioiodine and the efficacy of the iodine blockade is the nutritional supply with the diet (Robbins et al. 2001). It was reported that iodine deficiency may increase the risk of thyroid cancer induced by iodine-131 (Cardis et al. 2005). The recommended daily nutritional iodine intake is about 100–150 µg with higher requirements in particular situations (e.g., 200 µg in pregnant and lactating women). In some countries like Germany, the natural iodine deficiency in the diet (Iodine Global Network, 2020) is compensated by adding iodide to salt. Regional differences may exist as was shown in France, where the daily iodine supply decreases from western in eastern direction (Muller 2008). Japan on the other side, is a country with a very rich iodine diet, largely exceeding the recommended daily requirements (Ishigaki et al. 2001; Matsunaga and Kobayashi 2001; Takamura et al. 2004). Daily dietary intakes of several hundreds of µg have been repeatedly reported, e.g., 1523 ± 356 µg d^−1^ (Ohtaki et al. 1967) or 544 ± 321 µg d^−1^ (Katamine et al. 1986). It must, however, be acknowledged that nutritional habits may change over time so that the extremely high values sometimes reported must be viewed with caution. Nevertheless, nutritional differences may well influence the physiological state of the thyroid through physiological regulation mechanisms. It was stated that the “ICRP standard man” used to describe iodine kinetics for radiological purposes is not a valid model for the Japanese as reflected by a lower thyroidal uptake fraction (range 0.12–0.25) compared to Caucasians (0.3) (Yoshizawa et al. 1976). Based on an extensive literature search, Matsunaga and Kobayashi (2001) has estimated the kinetic parameters that should be used for Japanese in the classic iodine model of Riggs et al. (1952). However, these values were not used to simulate the consequences if applied to concrete scenarios. Moreover, the model of Riggs et al. does not permit to differentiate between the competition effect at the carrier site and the Wolff–Chaikoff effect, and is also not suited to simulate repetitive stable iodine administrations. We recently developed a new thyroid model based on an uptake mechanism described by a Michaelis–Menten kinetic and added an algorithm to describe the Wolff–Chaikoff effect and validated this model using empirical data (Rump et al. 2019). This model was also adapted as to permit simulations on thyroidal protection against radioiodine using perchlorate (Eder et al. 2020). In the present study, we adapted the parameters described for Japanese by Matsunaga and Kobayashi (2001) to our model and by simulations compared the sensitivity of the thyroid to radioiodine exposure as well as the protective efficacies of stable iodine and perchlorate in Caucasians and Japanese.

## Method

### The pharmacokinetic model for iodine

**Fig. 1:**
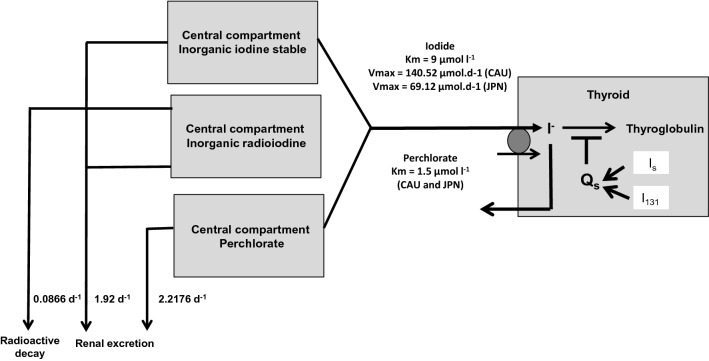
Compartment models for radioiodine and perchlorate with an integrated carrier uptake mechanism described by Michaelis–Menten kinetics for thyroidal iodide uptake. The competition of stable iodide and radioiodide at the carrier site is modeled by applying the rate law for monomolecular irreversible enzyme reactions to the transport mechanism. The Wolff–Chaikoff effect is modeled by a total thyroidal uptake block for iodine (lasting 36 h), starting when the gland is saturated (uptake amount *Q*_s_  + 350 µg iodine for Caucasians and  + 5000 µg for Japanese)

We previously developed a biokinetic model for iodine (Rump et al. 2019) derived from the model introduced by Riggs (1952) and used by the International Commission for Radioprotection (ICRP) (ICRP 1994, 1997) (Fig. 1). This model was previously validated by comparison with results obtained by the Integrated Modules for Bioassay Analysis (IMBA) software (Rump et al. 2019). In this two-compartment model, the central compartment represents the extracellular space including red blood cells and the second compartment represents the thyroid gland. As the absorption rates for iodine by inhalation as well as ingestion are very rapid and the extent almost complete (Geoffroy et al. 2000), we added iodine (radioiodine and stable iodine) directly into the central compartment. The elimination of iodine out of the body occurs from the central compartment by renal excretion that is not saturable and can be described by first order kinetics (rate constant: 1.9404 d^−1^). In the case of radioiodine only, an additional elimination out of the central compartment occurs by physical decay (T_1/2_ = 8 days, decay constant = 0.086625 d^−1^).

The transport of iodide from the central compartment into the thyroid is an active process mediated by the NI-symporter and thus cannot be described by first order kinetics in the presence of higher iodide concentrations as expected in the case of iodine blockade. That’s why for modeling iodide transport into the thyroid, we integrated a carrier mechanism described by Michaelis–Menten kinetics according to the formula:

$$ T = {{\left( {T_{\max } \times C} \right)} \mathord{\left/ {\vphantom {{\left( {T_{\max } \times C} \right)} {\left( {K_{{\text{m}}} + C} \right)}}} \right. \kern-\nulldelimiterspace} {\left( {K_{{\text{m}}} + C} \right)}} $$

with *T*: transport capacity into the thyroid; *T*_max_: maximum transport capacity; *K*_m_: Michaelis–Menten (affinity) constant; *C*: iodide concentration in the central compartment.

The kinetics of iodine integrated in thyroid hormones that is secreted from the thyroid into serum was not considered as it is a very slow process (rate constant 0.00866 d^−1^, T_1/2_ = 80 days) (Riggs 1952; ICRP 1989) compared to iodide uptake into the gland and our focus is on the latter process. In our model, the thyroidal compartment was considered as a sink.

To simulate a competition of stable iodine and radioiodine at the NI-symporter, in analogy we applied the rate law for monomolecular irreversible enzyme reactions with a number *i* of competing substrates (Chou and Talaly 1977; Schäuble et al. 2013):

$$ T_{{\text{i}}} = \frac{{T_{\max } \times C_{{\text{i}}} }}{{K_{{{\text{mi}}}} \times \left( {1 + \sum\nolimits_{{\text{i = 2}}}^{{\text{n}}} {\frac{{C_{{\text{i}}} }}{{K_{{{\text{mi}}}} }}} } \right) + C_{{\text{i}}} }} $$

with *T*_i_ the transport rate for substrate *i*, C_i_ the concentration of substrate i, *K*_mi_ the Michaelis–Menten constant for substrate i and *T*_max_ the maximum transport rate.

As described previously, we modeled the Wolff–Chaikoff effect by adding an additional saturation mechanism leading to a total thyroidal uptake block that is “switched on” when an iodine saturation amount has been reached (Rump et al. 2019) (Fig. 1). As the Wolff–Chaikoff effect is effective only temporarily (24–48 h) (Leung and Braverman 2014), this total uptake block was switched off after 36 h, so that only the competition at the carrier site remained active after this time point.

### The parameters of the iodine biokinetic models for Caucasians and Japanese

We assumed the volume of distribution of the central compartment to be identical in Caucasians and Japanese with approximately 16 l (water: 60% of body weight; extracellular space 1/3, i.e., 14 l and red blood cells about 2 l). We also assumed that the renal elimination of iodide is similar in Caucasians and Japanese and used the rate constant given by Matsunaga and Kobayashi (2001) (1.92 d^−1^ that only slightly differs from the value 1.9404 d^−1^ we used in our previous simulations) (Rump et al. 2019). As far as we know, there are no genetic polymorphisms for the NI-symporter in humans, and so we assumed the same Michaelis–Menten constant *K*_m_ = 9 µmol/l for Caucasians and Japanese. This is the value that has been given for humans (Darouzet et al. 2014) and that we used in our previous simulations (Rump et al. 2019; Eder et al. 2020).

The maximum transport capacity (*T*_max_) was derived from the *K*_m_ and the constant rate *k*_thyr_ describing the transport from the central compartment into the thyroid that is applicable when first order kinetics may be approximately assumed at very low iodide concentrations.

$$ T_{\max } = K_{{\text{m}}} \times k_{{{\text{thyr}}}} = 9 \times k_{{{\text{thyr}}}} $$

The rate constant *k*_thyr_ is given in the model used by Ramsden et al. (1967) to describe iodine blockade by the formula:

$$ k_{{{\text{thyr}}}} = f\left(1- {\frac{{Q_{{\text{t}}} }}{{Q_{{\text{s}}} }}} \right) $$

with *f* (d^−1^) a constant related to the transport from the central compartment to the thyroid gland; *Q*_t_ (mg): the average iodine content of the thyroid; *Q*_s_ (mg): the iodine content of the thyroid when the gland is saturated.

For Caucasians, we used the values proposed by Ramsden et al. (1967). There are only minor numerical differences to our previous model that was validated against the results given by the commercial software IMBA (Integrated Modules for Bioassay Analysis) (Birchal et al. 2007) for radioiodine exposure (Rump et al. 2019). For Japanese, we applied the corresponding values proposed by Matsunaga and Kobayashi (2001) that were revised with lower values for iodine saturation than previously reported by the same authors (Matsunaga and Kobayashi 2000). The latter parameters were derived from a survey of the literature with data on thyroidal iodine uptake and the iodine contents of the gland in Japanese. As the constants describing iodine uptake into the thyroid as well as iodine contents (average and at saturation) differ between Caucasians and Japanese, so do the maximum transport capacities for iodide into the gland. In our model, this is the only parameter showing a difference between the two groups. All values used to derive the parameters entered in our model are displayed in Table 1.

**Table 1:** Pharmacokinetic parameters for Caucasians (CAU) and Japanese (JPN) directly used in our model or that served to derive the maximum transport capacity of iodide uptake through the basolateral membrane of the thyrocytes described by Michaelis–Menten kinetics

Parameter	Symbol (unit)	CAU	JPN
Rate constant of the physical decay of I-131	*K*_phys_ (d ^−1^)	0.0866	0.0866
Rate constant of the renal elimination from the central compartment	*K*_ren_ (d^−1^)	1.92	1.92
Average iodine content of the thyroid	*Q*_t_ (mg)	8.00	15
Iodine content of the thyroid at saturation (total uptake block)	*Q*_s_ (mg)	8.35	20
Constant related to the transport from the central compartment to the thyroid for an empty gland (*Q*_t_ = 0)	*f* (d ^−1^)	23.28	1.92
Rate constant of the transport from the central compartment into the thyroid	*k*_thyr_ (d ^−1^)	0.9758	0.4800
Michaelis–Menten (affinity) constant of iodide for the NI-symporter	*K*_m_ (µmol)	9	9
Maximum iodide transport capacity from the central compartment into the thyroid	*T*_max_ (µmol.d^−1^)	140.52	69.12
Michaelis–Menten (affinity) constant of perchlorate for the NI-symporter	*K*_m_ (µmol)	1.5	1.5

Source of the data: Matsunaga and Kobayashi (2001) in part based on Ramsden et al. (1967) for Caucasians

### The pharmacokinetic model for perchlorate

The iodine biokinetic model described above has previously been extended to allow for simulations of thyroidal protection against radioiodine by perchlorate (Eder et al. 2020). An additional extracellular compartment for perchlorate was added to the iodine biokinetic model and renal elimination was modeled by first order kinetics. For perchlorate, we used the pharmacokinetic parameters reported in the literature (elimination rate constant 0.0924 h^−1^, half-life 7.5 h; volume of distribution 0.34 l kg^−1^, i.e., for a 70 kg adult 23.8 l) (Crump and Gibbs 2005; Lorber 2009). Similar to iodine and previous studies (Eder et al. 2020), we entered perchlorate directly into the central compartment in our simulations, as the oral absorption of perchlorate is rapid and complete (ATDSR 2008; BAuA 2016).

Similar to the competitive effect of stable iodine at the NI-symporter, we applied the rate law for monomolecular irreversible enzyme reactions with a number *i* of competing substrates (Chou and Talaly 1977; Schäuble et al. 2013) to describe the radioiodine uptake inhibition by perchlorate. As Michaelis–Menten (affinity) constant of perchlorate for the NI-symporter, we used 1.5 µmol/l (Kosugi et al. 1996) as it was shown that the lower value of 0.59 µmol/l (Schlosser 2016) for lower dose levels below 100 µg.kg^−1^ d^−1^ did not permit to reasonably simulate thyroid blocking with higher perchlorate dosages (Eder et al. 2020). We did not consider an uptake of perchlorate into the gland, and as reported in the literature, we assumed that perchlorate does not affect the organification process of iodide. This thyroid blocking model for perchlorate has been previously validated (Eder et al. 2020). The parameters for perchlorate were assumed to be identical for Caucasians and Japanese. The only difference between the two groups resides in different transport capacities for iodide through the membrane as described in the previous sections.

### Calculation of the thyroid equivalent dose from the radioiodine uptake into the gland

As previously described (Rump et al. 2019; Eder et al. 2020), the radiological thyroid equivalent dose was calculated by the Marinelli–Quimby method (1948) for the contribution of the β-radiation and by the geometrical factor method of Hine and Brownell (1956) for the (low) contribution of the γ radiation according to the formula:

$$ D \, = \, C_{\max } \times T_{{{\text{eff}}}} \times \left( {73.8 \times \overline{E}_{{\upbeta }} + 0.0346 \times T \times \overline{g}} \right) $$

With *D* the total dose from β and γ radiation (rad), *C*_max_ the maximum concentration of the radionuclide in tissue (µ*C*_i_ g^−1^), T_eff_ the effective half-life in the tissue (days) (7.3 days for I-131 in adults), $\overline{E}_{{\upbeta }}$ the average beta energy (MeV per disintegration) (0.18 MeV for I-131), Ƭ the specific γ ray constant (R per m*C*_i_ h^−1^ at 1 cm) (2.2 R m*C*_i_^−1^ h^−1^) and $\overline{g}$ the average geometrical factor for the tissue or organ, equal to 3 π *r* for spheres with radii < 10 cm) (*r* = 1.27 cm in adults assuming that the thyroid is made of two identical spheres of unit density) (values from National Cancer Institute 2015). The maximum concentration *C*_max_ was computed by dividing the maximum accumulated amount of iodine in the thyroid by the weight of the gland 17 g (National Cancer Institute 2015). This is a rather low estimate that is often exceeded in iodine-deficient regions. As we could not reasonably delimit thyroid weight differences between Caucasians and Japanese, we used the same value for calculations in both groups. All units were transformed to get the dose in mSv leading to the following formula:

$$ D\left( {{\text{mSv}}} \right) = 1.647 \times 10^{ - 3} \times {\text{accumulated I}} - {\text{131 in the thyroid }}\left( {{\text{Bq}}} \right) $$

Although quite ancient, this methodology is still widely used (Stabin et al. 2006; Spetz 2010; National Cancer Institute 2015). We did not consider the radiation emitted from other source organs to the thyroid as a target, as radioiodine mostly concentrates in the gland and iodine-131 mostly emits β-radiation. It must be mentioned that the doses we calculate are equivalent doses that must not be confused with effective doses that take into account the radiation sensitivity of a tissue regarding stochastic health effects (effective dose = equivalent dose × sensitivity factor, for the thyroid the sensitivity factor = 0.05; both dose concepts use the same unit Sv).

In all simulations involving thyroid blocking, the protective efficacy was determined as the complementary value of the quotient of the absorbed thyroidal equivalent dose with and without the blocking intervention (stable iodine or perchlorate administration):

$$ Efficacy \, = \, 1 - \left( { \, Equivalent \, dose \, with \, thyroid \, blocking/equivalent \, dose \, without \, thyroid \, blocking} \right) $$

In addition we computed the iodine uptake fraction as the quotient of the radioiodine amount accumulated in the thyroid relative to the amount entered into the body.

### Comparison of the equivalent thyroid doses in Caucasians and Japanese without thyroid blocking interventions

We determined the equivalent thyroid dose resulting from an acute radioiodine exposure (700,000 Bq) or a continuous radioiodine exposure (230,000 Bq/d) up to 10 days. The activities were chosen to be similar with previous studies (Rump et al. 2019) and at the time were determined as to slightly exceed the thyroid dose limit of 300 mSv according to German radioprotection regulations (in the case of continuous exposure after 3 days).

### Estimation of the protective efficacy of stable iodine or perchlorate at the recommended dosages

We determined the protective efficacy against acute radioiodine exposure (700,000 Bq) of a single dose of stable iodine at the recommended dosages of 100 mg (recommendations in Germany) for Caucasians or 76 mg (recommendations in Japan) for Japanese. Stable iodine was administered simultaneously with acute radioiodine exposure. Protective efficacy was determined considering only the competition mechanism at the NI-symporter or taking into account in addition the Wolff–Chaikoff effect to quantify the relative contribution of the latter to the overall thyroidal protection.

Perchlorate was administered at a dosage of 1000 mg in Caucasians and Japanese, as this dose is usually considered to be equivalent to 100 mg stable iodine (Hänscheid et al. 2011; Eder et al. 2020).

Protective efficacies were calculated based on the total accumulated amount of radioiodine in the thyroid at the end of the 1st or the 10th day after acute radioiodine exposure.

### Comparison of the median protective doses of stable iodine and perchlorate in the case of acute radioiodine exposure

The equivalent thyroid dose resulting from an acute single intake of radioiodine (700,000 Bq) was determined when administering different doses of stable iodine or perchlorate at the same time as radioiodine exposure. Radioiodine as well as stable iodine or perchlorate were entered directly into the central compartment and efficacy calculations were again based on radioiodine accumulation up to 1 day or 10 days after exposure.

The dose–effect relation was determined by fitting the data to a sigmoidal Hill equation with three parameters $\left( {E = \frac{{a \times D^{{\text{b}}} }}{{D50^{{\text{b}}} + D^{{\text{b}}} }}} \right)$ with *E*: the relative effect between 0 and 1; a: the maximum relative effect set at 1; *D*: the iodine dose; *D*_50_: the iodine dose leading to a half maximum effect *E* = 0.5 (i.e., the median protective iodine dose); *b*: the Hill coefficient reflecting the steepness of the linear part of the curve.

### Estimation of the protective efficacy of stable iodine or perchlorate in the case of continuous radioiodine exposure

In a further simulation, we determined the protective efficacy of single or repetitive daily doses of stable iodine (76 mg or 100 mg) or of perchlorate (1000 mg) in the case of a continuous radioiodine exposure (230,000 Bq/d) lasting for 10 days. Thyroid blockade was started simultaneously with the beginning of radioiodine exposure. For stable iodine, we considered the protective effect by competition at the carrier site only (i.e., without Wolff–Chaikoff effect) or taking into account a total iodine uptake block by the Wolff–Chaikoff effect lasting for 36 h (Leung and Braverman 2014; Eder et al. 2020). In all cases, protective efficacy was calculated based on the total radioiodine accumulated up to the end of the 10th day after the beginning of radioiodine exposure.

## Results

### Comparison of the equivalent thyroid doses in Caucasians and Japanese without thyroid blocking interventions

**Fig. 2:**
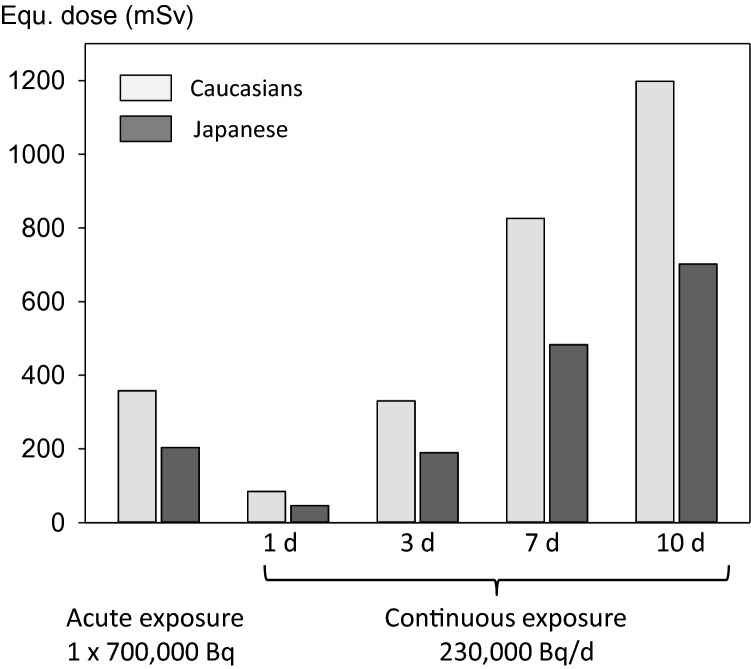
Thyroid equivalent doses in Caucasians and Japanese caused by an acute radioiodine exposure of 700,000 Bq or a continuous exposures of 230,000 Bq/d lasting for different periods up to 10 days. Calculations are based on radioiodine accumulated in the thyroid 1 day after acute exposure or at the end of the exposure period in the case of continuous exposure

Acute radioiodine exposure (700,000 Bq) leads to a thyroid equivalent dose of 358 mSv with an iodine uptake fraction of 0.31 in Caucasians based on radioiodine accumulated in the gland after 1 day. The values are slightly larger than in similar previous simulations for the same radioactivity exposure (358 mSv vs. 316 mSv) (Rump et al. 2019) as the maximum transport capacity used in the Michaelis–Menten kinetics is derived from the first order rate constants for low iodide concentrations that slightly differ in the studies (0.9758 d^−1^ as indirectly derived from the data from Ramsden et al. (1967) vs. 0.8316 d^−1^ in our previous studies). In Japanese, the same exposure leads to a substantially lower equivalent dose of 204 mSv with an uptake fraction of 0.18 (Fig. 2).

In the case of continuous radioiodine exposure (230,000 Bq/d), the thyroid equivalent doses increase with exposure time and are less in Japanese compared to Caucasians (e.g., after 10 days, 1,198 mSv in Caucasians vs. 702 mSv in Japanese) (Fig. 2). The uptake fractions also increase from the 1st to the 10th day of exposure (from 0.223 to 0.316 in Caucasians and from 0.122 to 0.185 in Japanese).

### Estimation of the protective efficacy of stable iodine or perchlorate at the recommended dosages

**Fig. 3:**
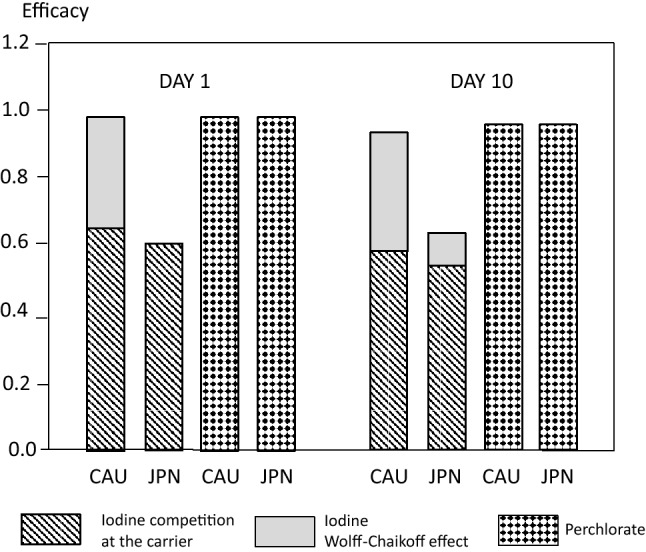
Protective efficacy of thyroid blocking against acute radioiodine exposure by a single administration of stable iodine in the officially recommended dosage (100 mg in Germany, 76 mg in Japan) or perchlorate (1000 mg). The thyroid blocking agent is given simultaneously to the acute radioiodine exposure. For thyroid blocking by stable iodine, the contribution of the competition at the NI-symporter and the Wolff–Chaikoff effect is shown

At the officially recommended dosages, a single dose of stable iodine is more effective in Caucasians (100 mg, efficacy 0.9887 after 1 day) than in Japanese (76 mg, efficacy 0.6433) (Fig. 3). Although the effect achieved by competition at the NI-symporter is quantitatively quite similar (efficacy about 0.6), the contribution of the Wolff–Chaikoff effect in Japanese is almost absent after 1 day (2.2% of the total efficacy) and only weak after 10 days (15.1%). On the contrary, the Wolff–Chaikoff effect contributes to about 35% to thyroidal protection in Caucasians (Fig. 3). According to our simulations, this results from the fact that the iodine saturation of the gland leading to a total thyroid uptake block becomes effective much faster in Caucasians at a time when iodide concentrations are still high in the central compartment (Table 2). Even if enhancing iodine dosage from 76 to 100 mg, it would confer less thyroidal protection in Japanese than in Caucasians (efficacy 0.7265 instead of 0.6433 in Japanese vs. 0.9887 in Caucasians).

**Table 2:** Onset time of a total uptake block of the thyroid by the Wolff–Chaikoff effect in Caucasians (CAU) and Japanese (JPN) depending on the dose of stable iodine

Iodine dose (mg)	Onset time	
	CAU (min)	JPN
5	146.59	> 10 d
25	49.82	> 10 d
50	38.88	1.56 d
76	35.14	22.69 h
100	33.55	19.73 h
200	30.82	16.26 h
500	29.38	14.63 h
1000	28.80	15.15 h

At low doses, saturation is achieved in Japanese at a late time point without relevant radioiodine concentrations in blood and so the Wolff–Chaikoff effect does not contribute to the protective efficacy

**Fig. 4:**
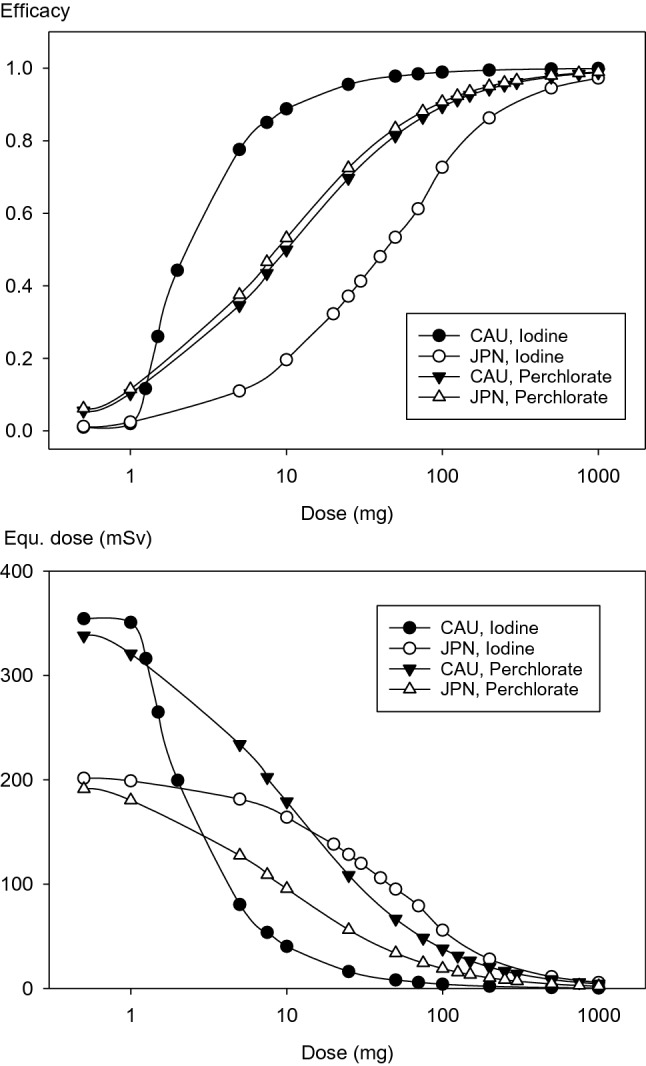
Dose effect curves for stable iodine and perchlorate administered simultaneously with an acute radioiodine exposure (700,000 Bq). The Wolff–Chaikoff effect is assumed to last at least for 24 h. The calculations are based on the amounts of radioiodine accumulated up to 24 h after exposure. The upper figure shows the (relative) efficacy = 1 − (thyroid equivalent dose with blockade/thyroid equivalent dose without blockade). The bottom figure shows the equivalent doses (mSv) absorbed by the thyroid taking into account the lower dose absorbed without thyroid blocking in Japanese compared to Caucasians

According to our definition, efficacy is a relative measure based on the dose reduction factor (dose with thyroid blocking/dose without thyroid blocking) and it does not take into account the absolute values of the radiological dose (mSv). However, in Japanese, thyroid equivalent doses without protective treatment are lower than in Caucasians (204 mSv in Japanese vs. 358 mSv in Caucasians after 1 day). Nevertheless, at the recommended stable iodine doses, the equivalent thyroid dose absorbed in Japanese (with 76 mg: 72.77 mSv) is higher than in Caucasians (with 100 mg iodine: 4.03 mSv) (Fig. 4).

Thyroid blocking by perchlorate confers the same protection in Caucasians and Japanese (0.9878 and 0.9894, respectively) corresponding to the efficacy achieved by 100 mg stable iodine in Caucasians (0.9887) (Figs. 3 and 4).

### Comparison of the median protective doses of stable iodine and perchlorate in the case of acute radioiodine exposure

The calculated thyroidal protection increases concomitantly with the amount of single doses of stable iodine or perchlorate administered simultaneously to acute radioiodine exposure (Fig. 4). With regard to the efficacy values based on accumulated radioiodine amounts in the gland, no substantial differences can be observed 1 day or 10 days after exposure.

Despite the differences in parameters between the model for Caucasians and Japanese (maximum transport capacity for iodine), the dose–effect curves for perchlorate run parallel and close to each other with small numerical differences for the potency (ED_50_ = 10.04 mg in Caucasians and 8.74 mg in Japanese after 1 day) (Table 3). In both groups, a very high efficacy is achieved with 1000 mg perchlorate (0.988 in Caucasians, 0.989 in Japanese, a dose that is usually considered as equivalent to 100 mg iodine) (Hänscheid et al. 2011; Eder et al. 2020) (Fig. 4).

**Table 3:** Median effective doses (ED_50_) and Hill coefficients of the protective efficacy of stable iodine or perchlorate after an acute radioiodine exposure in Caucasians (CAU) and Japanese (JPN)

	Iodine without WC effect		Iodine with WC effect		Perchlorate	
	CAU	JPN	CAU	JPN	CAU	JPN
ED_50_ (mg) 1 day	52.75	43.44	2.71	41.60	10.04	8.74
Hill coeff	0.9723	0.9620	2.0168	1.0512	0.9291	0.9344
ED_50_ (mg) 10 days	67.28	56.02	2.84	46.15	13.54	11.62
Hill coeff	0.8719	0.8703	1.7286	1.222	0.7887	0.8174

Results are given with and without taking into account the Wolff–Chaikoff (WC) effect assumed to last at least for 24 h. Calculations are based on the radioiodine activity accumulated in the thyroid after 1 or 10 days after radioiodine exposure

In the case of thyroid blocking by stable iodine, taking into account the Wolff–Chaikoff effect, the dose–effect curve shows a greater steepness, reflected in a higher Hill coefficient (2.01 vs. 1.05) for Caucasians compared to Japanese. Concomitantly, stable iodine has a higher potency for thyroid blocking in Caucasians than in Japanese (ED_50_ = 2.71 mg vs. 41.60 mg after 1 day) (Table 3). According to our simulation results, this is caused by the Wolff–Chaikoff effect becoming effective earlier in Caucasians, as explained in the previous section on efficacy achieved by the recommended iodine dosages.

At very low iodine or perchlorate dosages, the absolute equivalent thyroid doses remain lower in Japanese (1 mg stable iodine: 199 mSv in Japanese vs. 351 mSv in Caucasians after 1 day) (Fig. 4). The curves for perchlorate converges for higher dosages and at 1000 mg there is no clinically relevant difference between the two groups as mentioned in the previous sections (4.37 mSv in Caucasians vs. 2.15 mSv in Japanese). The dose–effect curves for stable iodine taking into account the Wolff–Chaikoff effect also converges to the right, but at the same dosages the spread between the curve for Caucasians and Japanese is larger than for perchlorate (Fig. 4).

### Estimation of the protective efficacy of stable iodine or perchlorate in the case of continuous radioiodine exposure

**Fig. 5:**
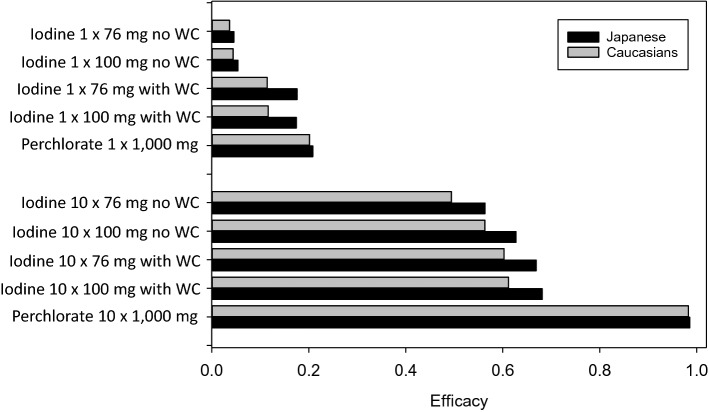
Protective efficacy by single or repetitive daily doses of stable iodine at the officially recommended dosages (100 mg in Germany or 76 mg in Japan) or perchlorate (1000 mg) in the case of continuous radioiodine exposure for 10 days in Caucasians (CAU) and Japanese (JPN). The first dose is given at the time exposure starts. Protective efficacy is based on the amounts of radioiodine accumulated at the end of the 10th day. Efficacy = 1 − (thyroid equivalent dose with blockade/thyroid equivalent dose without blockade). *WC* Wolff–Chaikoff effect assumed to last 36 h after the thyroid gland is saturated with iodine

In the case of continuous radioiodine exposure for 10 days, a single dose of stable iodine at the recommended dosages offer a substantially lower thyroidal protection than for acute radioiodine exposure (Fig. 5, Table 4). At the opposite of acute radioiodine exposure, efficacy is nevertheless numerically higher in Japanese (76 mg, efficacy 0.1757) compared to Caucasians (100 mg, 0.1163), probably because of the lower maximum transport capacity combined with the limited effectiveness of the Wolff–Chaikoff effect over time. A marginally better but still unsatisfactory efficacy can be achieved by a single dose of 1000 mg perchlorate (efficacy 0.20). The absolute values of the equivalent thyroid doses are nevertheless substantially lower after a 10 day radioiodine exposure in Japanese compared to Caucasians (1000 mg perchlorate once, 555.7 mSv in Japanese, 956.9 mSv in Caucasians).

**Table 4:** Protective efficacy of single or repetitive daily doses of stable iodine at the officially recommended dosages (100 mg in Germany or 76 mg in Japan) or perchlorate (1000 mg) in the case of continuous radioiodine exposure for 10 days in Caucasians (CAU) and Japanese (JPN)

	Single dose		Repetitive daily doses	
	CAU	JPN	CAU	JPN
Iodine 76 mg no WC	0.0365	0.0453	0.4940	0.5636
Iodine 100 mg no WC	0.0440	0.0537	0.5632	0.6274
Iodine 76 mg WC 36 h	0.1143	0.1757	0.6027	0.6690
Iodine 100 mg WC 36 h	0.1163	0.1744	0.6121	0.6815
Perchlorate 1000 mg	0.2012	0.2082	0.9829	0.9856

The first dose is given at the time exposure starts. Protective efficacy is based on the amounts of radioiodine accumulated at the end of the 10th day. Efficacy = 1 − (thyroid equivalent dose with blockade/thyroid equivalent dose without blockade). *WC* Wolff–Chaikoff effect assumed to last 36 h after the thyroid gland is saturated with iodine

Efficacy can be much improved by repetitive daily doses of iodine or perchlorate (Fig. 5, Table 4). With repetitive daily doses of stable iodine at the officially recommended dosages, in Japanese it is possible to achieve roughly the same efficacy as in acute radioiodine exposure with a single dose. However, this confers only a partial protection (76 mg, efficacy 0.6690). In Caucasians, protective efficacy is numerically lower than in Japanese but in a similar order of magnitude (100 mg, efficacy 0.6121). The high protective effect of a single iodine dose in acute radioiodine exposure (0.9887) based on a rapid onset of a total uptake block when the gland is saturated is not achieved in the case of continuous exposure as the Wolff–Chaikoff effect is only temporary.

The best protective effect similarly in Caucasians and Japanese (0.9829 and 0.9856, respectively) with a level comparable to thyroid blocking in the case of acute radioiodine exposure is achieved with 1000 mg of perchlorate (Fig. 5, Table 4).

## Discussion

Regional differences in the effectiveness of drug therapy can be observed and may be due to differences in the pharmacokinetics and or pharmacodynamics of the pharmacological agent in different populations (Woodworth et al. 2004). Metabolic inter-ethnic differences have been reported for phase I as well as phase II enzymes: Reduced activity of the cytochrome Cyp2D6 is found in 5–10% of Caucasians, 6–8% of Afro-Americans, but only 1% of Asians (Montay et al. 2011). On the other side, among Caucasians less than 1% have been shown to have a low cytochrome 2A6 activity, whereas among Orientals (Japanese and Chinese) up to 20% are poor metabolizers (Raunio et al. 2001; Montay et al. 2011). It is also well known that alcohol and aldehyde dehydrogenases exhibit genetic heterogeneity leading to differences in the effects of alcoholic beverages. Besides these “intrinsic factors” dependent on genetic differences, environmental factors (“extrinsic factors”) like differences in lifestyle, nutritional habits, but also medical practice may substantially influence therapeutic results (Naito 2000). Regional differences must be accounted for in drug development and despite international harmonization rules introduced in the nineties to facilitate drug approval processes in foreign countries, local authorities may ask for the results of “bridging studies” before granting marketing authorization (Liu et al. 2002; Woodworth et al. 2004; FDA 2006). Large differences in the daily iodine intake between Caucasians of several European countries and Japanese is an “extrinsic factor” that may cause differences in thyroidal function and the response to iodine blockade. However, as far the authors are aware, no “bridging study” has been done to assess the effect of large doses of stable iodine to protect the gland against radioiodine, so that up to now expected differences could not be described quantitatively.

Iodide is transported actively from serum into the thyrocytes by a sodium–iodide symporter (NIS) located in the basolateral membrane of the cells. Transport is secondary to the sodium gradient that is built up by the Na^+^/K^+^-ATPase in the membrane (Ferreira et al. 2005). The activity of NIS is regulated by thyrotrophin (TSH) that stimulates the expression of the NIS gene and thus enhances iodide uptake into the gland. In addition, NIS is found in intracellular vesicles that can possibly be rapidly mobilized to increase NIS activity in the basolateral membrane under the influence of TSH (Ferreira et al. 2005). Besides TSH, there is an autoregulation of thyroid function by intracellular iodide that at higher concentrations reduces iodide transport through the membrane and iodide organification in vivo as well as in vitro (Ferreira et al. 2005). The precise mechanisms involved in this Wolff–Chaikoff effect are not yet fully elucidated, but the effect could possibly be mediated by organic iodoaldehydes (Ferreira et al. 2005) and a reduction of peroxidase activity, leading to an inhibition of the hormonal synthesis and integration of iodine into thyroglobulin (Wolff 1969; Leung and Braverman 2014). This is associated with an increase of the free intracellular iodide concentration. The rise of the gradient between the intra- and extracellular space will accelerate the amount of iodide flowing back out of the cells by passive diffusion, as thyrocytes express also transporters and channels common to many epithelia and iodide may cross membranes using the same ways as chloride (Simchowitz 1988; Fong 2011). This is consistent with some models of iodide trapping that include a pathway out of the follicular cells into plasma described by first order kinetics (Hays 1979; Bazin et al. 1981; Merril et al. 2005). This enhanced passive outflow of inorganic iodide out of the thyrocytes may be expected to contribute to the protection of the gland against radioiodine in thyroid blocking. The Wolff–Chaikoff effect is, however, only transient (24–48 h) (Geoffroy et al. 2000).

Realistic modelling of the competition at the membrane carrier can be done using Michaelis–Menten kinetics as the Michaelis–Menten constants (*K*_m_) of the human NI-symporter for iodide and perchlorate have been determined and reported in the literature. However, modelling the Wolff–Chaikoff effect for iodine, as long as the underlying mechanisms are speculative, is a challenge. We postulated a total net uptake block for iodide that is “switched on” when the saturation level in the gland is reached for a limited and defined period of time (36 h), and then “switched off”. We used the saturation values reported by Ramsden et al. for Caucasians (+ 0.350 mg) and the values given by Matsunaga (2001) for Japanese (+ 5 mg). The large difference between these two saturation values, as well as the differences in the range of total iodine content of euthyroid glands reported in different regions in the world, already indicate that iodide uptake regulation is presumably not based on absolute saturation values but rather on a derived cybernetic variable. Therefore, our Wolff–Chaikoff modelling should not be viewed mechanistically, although our simulation results reflect well empirical data on the efficacy of iodine blockade (Rump et al. 2019). Modelling of thyroidal protection by perchlorate is much less problematic than for iodine as competition at the carrier site is the only mechanism involved.

It seems that in the “standard Japanese” with a high nutritional daily iodine intake, the transport capacity through the basolateral membrane is down regulated compared to the “standard Caucasian”, as to limit the iodide uptake into the gland to an adequate physiological amount (Matsunaga and Kobayashi 2001). This substantially lower iodide uptake fraction confers the Japanese a better natural protection against radioiodine, in the case of an acute as well as continuous radioiodine exposure. It must nevertheless be mentioned that we derived the parameters for our simulations from the constants given by Matsunaga and Kobayashi (2001) for a standard Japanese resulting in a rate constant for the transport of iodide into the thyroid of *k* = 0.48 d^−1^. This value, however, shows a large variability (range for *k* 0.3–1.2 d^−1^) and thus, the radiological doses may also substantially vary.

For the calculation of the equivalent dose, we assumed a thyroid weight of 17 g. This value is considered as a reasonable value averaged for both sexes in the US by the US National Cancer Institute (2015) and based on extensive autopsy examinations: e.g., New York, *n* = 762, mean 17.5 g in males, 14.9 g in females (Mochizuki et al. 1963); Seattle, *n* = 1,400, 18.5 g in males, 14.4 g in females (Pankow et al., 1985). These values are lower than reference weights given in the ICRP publication 23 (1975). There is, however, a great variability and in individual studies significantly higher weights were determined both in the US and in particular in Europe: Cleveland (in the Midwest, “goiter belt”), *n* = 408, 26 g for males, 25 g for females (Hazard et al. 1952); Jutland, Denmark, 25.5 g for males, 22.9 g for females (Agerbaek et al. 1974). In a cohort of more than 4000 people in Northeastern Germany (Pomerania), the mean thyroid volume was determined with 21.79 ml for males and 15.31 ml for females (Katthak et al. 2016). The prevalence of goiter in this region amounted to roughly 35%. Regional iodine deficiencies are an important determining factor. Thus, because of general biometric differences and the high nutritional iodine supply, a lower thyroid weight could be expected for the “standard Japanese”. In fact, a slightly lower weight was specified in the model for the “Reference Asian Man” (“Tanaka man”, 19.0 g for males, 16.9 g for females) (Tanaka, 1998) than for the “ICRP Reference Man” (Caucasian) (20 g for males, 18 g for females) (ICRP 1975). However, all these values are above the value of 17 g reported as the best estimate for the US. A review of individual studies of the thyroid weights of Japanese confirms mainly lower values than in iodine deficient world regions: mean values from 15.96 to 19.07 g for males and 14.30–17.61 g for females (Yoshizawa et al. 1976). Considering the range of values, it did, however, not seem possible to us to reasonably delimit the thyroid weights of Japanese and Caucasians, and that’s why we used a uniform value (17 g) for our dosimetric calculations.

Notwithstanding the large physiological variability and uncertainties, the 24 h iodide uptake fraction calculated with our model (12.2%) is very similar to the value experimentally determined in Japanese volunteers without dietary iodine restrictions (median 13%, range 5–26%) (Kunii et al. 2016). However, despite a lower absorbed radiological dose in Japanese compared to Caucasians, thyroidal protection by an iodine rich diet is still incomplete and thyroidal blockade is indicated in case of a radiological emergency (Takamura et al. 2003, 2004).

It was hypothesized that a lower dose of stable iodine may possibly be sufficient to achieve an effective protection of the thyroid exposed to radioiodine in Japanese (Kunii et al. 2016). Our results show a quite complex picture: although the thyroid is better protected in Japanese because of the lower transport capacity through the membrane, it seems that Caucasians react with a much higher sensitivity and, therefore, much more rapidly to an iodine saturation of the gland. Whereas a total iodide uptake block through the Wolff–Chaikoff effect becomes effective within the first hour after the recommended dose for iodine blockade in Caucasians (33 min after 100 mg iodine), this protective mechanism is activated only after several hours in Japanese (22.7 h after 76 mg iodine), limiting thyroidal protection. In the case of an acute radioiodine exposure, this is reflected by a higher protective potency of stable iodine in Caucasians (ED_50_ = 2.71 mg) compared to Japanese (ED_50_ = 41.6 mg) when using the radiological dose reduction factor to calculate efficacy. After acute radioiodine exposure, the protective efficacy of a dose of 100 mg stable iodine amounts to 0.989 after 1 day in Caucasians, but for a dose of 76 mg stable iodine only to 0.643 in Japanese. Increasing iodine dosage to 100 mg in Japanese would enhance efficacy (0.727) without reaching the protection conferred to Caucasians. Protective efficacies calculated for Caucasians using our model are in the same order of magnitude as values reported in the literature (Blum et al. 1967; Geoffroy et al. 2000). Empirical measurement results in Japanese show a quite large variability. For Japanese volunteers, the protective effect of 76 mg stable iodine has been reported to amount to 0.795 at 24 h after the administration of iodine-123 (Takamura et al. 2004). It must, however, be mentioned that the volunteers suffered of hyperthyroidism with a relatively high baseline iodine uptake of 44.5%. On the other side, it was reported that in euthyroid Japanese volunteers a low dose of 10 mg stable iodine given 1 h before iodine-123 administration led to a median thyroidal uptake inhibition of 81% after 24 h showing, however, a large range from 0 to 92.3% (Kunii et al. 2016). It should nevertheless be remembered that efficacy is not an absolute but a relative value and the radiological doses absorbed without protective treatment are substantially lower in Japanese for the same level of acute radioiodine exposure.

In the case of a continuous exposure, the situation is different, as the Wolff–Chaikoff effect is a temporary phenomenon and contributes less to thyroidal protection than in an acute exposure. Following an exposure for 10 days, single or repetitive doses of stable iodine confers a slight numerical advantage in efficacy to the Japanese. This finding can easily be explained by the lower transport capacity through the membrane for iodide in Japanese. However, considering the range of individual variability, the differences in the protective efficacy of 100 mg iodine in Caucasians (given daily, efficacy: 0.612) or 76 mg iodine in Japanese (given daily, efficacy 0.669) appears rather marginal and not of clinical relevance. As already demonstrated in previous analyses (Rump et al. 2019; Eder et al. 2020), in the case of continuous radioiodine exposure, a single dose of iodine is insufficient (efficacies in a range of 10–20%) and repetitive doses are of great importance to achieve good protective efficacy.

Although stable iodine is usually considered as the standard for thyroidal protection against radioiodine (WHO 2017; SSK 2018), perchlorate can be considered as an alternative, provided it is administered at equieffective dosages (1000 mg perchlorate are as effective as 100 mg stable iodine in acute radioiodine exposure) (Harris et al. 2009; Hänscheid et al. 2011; Eder et al. 2020). Perchlorate protects the thyroid by competition with radioiodine at the NI-symporter site showing even a lower Michaelis–Menten constant than iodine (Km 1.5 µmol/l vs. 9 µmol/l) (Kosugi et al. 1996), but has been reported to leave the organification of iodine unaffected. Our results show that the efficacy of perchlorate is roughly identical in Caucasians and Japanese in the case of acute as well as continuous radioiodine exposure. For continuous radioiodine exposure, 1000 mg achieves a better protection than 100 mg stable iodine, both agents given daily over 10 days. This can be easily explained by its higher affinity for the NI-symporter compared to iodine. Besides its good protective efficacy, it could be advantageous in particularly vulnerable populations as pregnant or lactating women, as the still immature thyroids of the fetus or newborn are particularly prone to a longer lasting Wolff–Chaikoff effect with a risk of hypothyroidism impairing neurocognitive development (Sun et al. 2009; Jourdain et al. 2010; Connelly et al. 2012).

Perchlorate has been used for the treatment of hyperthyroidism with minor side effects that were found to be less than when using thionamide drugs (Krüskemper et al. 1960, 1962). Its use became very limited after the occurrence of seven cases of fatal aplastic anemia in the 1960’s. The mechanisms of this serious adverse effect are still unknown (Wolff 1998), but as 4 of 7 cases occurred in a cluster, a contamination of the badges has been discussed as a possible cause. It should be mentioned that thionamides, as another important class of antithyroid agents, are also known to cause potentially life-threatening side effects like agranulocytosis (Cooper 2005; Bukhari et al. 2017) or aplastic anemia (Escobar-Morreale et al. 1997; Yamamoto et al. 2004). Meanwhile, there is a resurgence of perchlorate use, in particular for the treatment of amiodarone induced thyroid dysfunction, and no serious side effects have been reported (Wolff 1998; Suwansaksri et al. 2018). Thus, there is no scientific evidence precluding a short term use of perchlorate for thyroid blocking. In Germany, perchlorate is officially approved for the initiation of hyperthyroidism treatment (Irenat^®^ 300 mg perchlorate/ml = 15 drops; for adults 800–1000 mg/d in the first 1 to 2 weeks, in special cases up to 1500 mg/d, thereafter median daily dose 400 mg/d) or thyroidal protection in case of scintigraphy examinations of other organs using radioiodine (200–400 mg, in individual cases up to 1000 mg) or the perchlorate discharge test (600–1000 mg) (Gelbe Liste 2019). The recommended dosages are in the range that confers the same protection against radioiodine exposure as 100 mg stable iodine. However, perchlorate medication is not available in all countries and in particular not in Japan or the US (in the US, the marketing of Perchloracap^®^ with 200 mg perchlorate/capsule has been discontinued) (Reference.md 2020), and perchlorate in drinking water and some foods is rather perceived as an environmental issue (Sellers et al. 2007; Maffini et al. 2016).

## Conclusion

Our results confirm that nutritional habits with differing daily iodine intake represent an important extrinsic factor affecting the regulation mechanisms of thyroidal function. The iodine rich diet seems to down regulate the transport capacity for iodide through the basolateral membrane of the thyrocytes and confers the Japanese a “natural” protection against radioiodine compared to Caucasians, although protection is nevertheless inadequate and thyroidal protection still indicated. Our results, however, also indicate an increased thyroidal sensitivity to iodine saturation (Wolff–Chaikoff effect) in Caucasians compared to Japanese, resulting in a higher efficacy of iodine blockade against acute radioiodine exposure. In the case of continuous radioiodine exposure and in parallel to a fading Wolff–Chaikoff effect, iodine blockade is marginally more effective in Japanese, although the differences do not seem to be of clinical relevance over the range of recommended iodine dosages. In such a scenario repetitive daily dosages are of major importance for the protection of the gland. Optimum thyroidal protection in case of acute or continuous radioiodine exposure can be achieved by administration of perchlorate instead of stable iodine in both, Caucasians and Japanese. Considering its simpler protective mechanism, potential advantages in particularly vulnerable subpopulations and its acceptable adverse effects, it seems promising for future studies to focus more closely on perchlorate as an alternative to stable iodine for thyroidal protection against radioiodine.
